# Community participation in malaria control strategy of intersect oral collaboration in Ghana: Myth or reality?

**DOI:** 10.4102/phcfm.v6i1.467

**Published:** 2014-08-01

**Authors:** Nicodemus O. Owusu

**Affiliations:** 1 Faculty of Human and Social Sciences, University of Southampton, United Kingdom

## Abstract

**Background:**

For many years, malaria has been one of the main health concerns of the government of Ghana. The government has recently implemented a control strategy which will ensure the inclusion of the community members who were previously excluded from the process. Until now, however, scientific study on this strategy has been scanty.

**Objectives:**

The objectives were to investigate the level at which communities have been allowed to participate and to understand whether the idea of community participation in malaria control strategy is a myth or a reality.

**Methods:**

Data were collected in the rural district of Ahafo-Ano South in the Ashanti region of Ghana. An exploratory qualitative approach was employed in order to ascertain the opinions of the local health officials and community members. The level of participation was measured using the framework of Arnstein's ‘ladder’ of participation, as developed in 1969.

**Results:**

Evidence showed that the level of community participation was only tokenistic. Communities were only informed and/or consulted after decisions had been made, but the real engagement and negotiations were absent. Communities thus had limited opportunities to air their views in the planning process.

**Conclusion:**

This article has revealed that the government's vision of ensuring community participation in the malaria control policy-making process can be said to be a myth rather than a reality.

## Abstrait

**La participation communautaire dans la stratégie de lutte antipaludique de collaboration orale au Ghana: mythe ou réalité?**

**Contexte:**

Le paludisme a été, pendant de nombreuses années, l'une des principales préoccupations du gouvernement du Ghana en matière de santé. Le gouvernement a récemment mis en œuvre une stratégie de contrôle qui assurera l'inclusion des membres de la communauté qui étaient jusqu'ici exclus du processus. Cependant, les études scientifiques sur cette stratégie ont été rares jusqu'ici.

**Objectifs:**

Les objectifs étaient d'étudier le taux de participation des communautés et de déterminer si l'idée de participation de celles-ci à la stratégie de contrôle du paludisme était un mythe ou une réalité.

**Méthodes:**

Les données ont été collectées dans le district rural d'Ahafo-Ano-Sud dans la région Ashanti du Ghana. Une approche exploratoire qualitative a été employée pour déterminer les opinions des responsables de la santé et des membres de la communauté. Le taux de participation a été mesuré à l'aide de «l'échelle» de participation d'Arnstein, développée en 1969.

**Résultats:**

Les résultats ont montré que le niveau de participation de la communauté n'était que symbolique. Les communautés n'avaient été informées et/ou consultées qu'après la prise des décisions, et il n'y avait pas eu d'engagement ni de négociations véritables. Les communautés n'avaient eu que peu d'opportunités d'exprimer leurs points de vue dans le processus de planification.

**Conclusion:**

Cet article montre que la vision du gouvernement de faire participer la communauté au processus d'élaboration d'une politique de contrôle du paludisme est un mythe plutôt qu'une réalité.

## Introduction

### Setting

For more than a century now, malaria has been one of the key health issues confronting the government of Ghana. The disease has become one of the country's sources of underdevelopment as a result of economic losses and a high rate of morbidity and mortality.^1,2,3^ Available evidence shows that about 44% of all outpatient illnesses caused by malaria are treated annually in the government hospitals.^3,4^ In addition, it is estimated that 36% of all those admitted at the hospitals and more than 22% of all children under five years of age who die every year can be attributed to malaria.^4,5^ With regard to pregnant women, it has been noted that over 13.8% are infected with malaria, with 9.4% of all maternal deaths being caused by the disease.^4,5^ Many commentators have argued that these figures are merely the tip of the iceberg since most cases are not reported because they are dealt with at home.^6,7^ In terms of economic losses, malaria poses a great financial burden on both households and the economy.^1,8,9^ For example, a study has shown that a single episode of malaria in a household can result in an estimated average cost of almost 13.4 new Ghana Cedis ($15.79)^1,8^, whilst nationally, just a 1% increase in the malaria morbidity rate might reduce the rate of real gross domestic product (GDP) growth by 0.41%.^8,9^

Faced with these problems, the last 10 years have seen a commitment from the government to address the problem by establishing an intersectoral collaboration strategy (ISC) that will allow grassroots members to be included in the malaria control policy-making process.^6,10,11^ Central to the thinking behind this strategy is that by allowing the communities to participate, members would be empowered to have ownership of programme activities, could accept the challenges associated with the control of the disease and, above all, could contribute more effectively to the success of the policy goal of minimising the persistence of malaria in the country.^12,13,14^ However, until now, very little has been known about the level of community participation in the ISC strategy for the control of malaria in Ghana. This makes it difficult to know whether this noble collaborative initiative in the country is rhetoric or a reality. This article therefore aims to assess how far communities have really been allowed to participate in control policy-making process in the rural district of Ahafo-Ano South (AAS) in the Ashanti region of Ghana.

The article begins with an explanation of the concept of community participation followed by Arnstein's famous ‘ladder’ framework.^15^ It then tries to analyse which position the study site will occupy on the participation ladder. The article concludes by arguing that simply having communities play a role in the implementation process is not enough – it is more about allowing them to have the necessary influence on the planning process.

### Significance of the study

Whilst participation in Ghana has been promoted in the health sector for more than a decade, most studies on the subject, until now, have mostly centred on political issues. There is therefore a paucity of information regarding community participation in the health sector. This makes this study important, when taking into account the timing, the sector and the place where the study was conducted. With respect to the timing, the study was conducted after Ghana had not only signed up to the Roll Back Malaria Agenda and become the recipient of Global Health Funds, but had also received international recognition of being one of the most democratic countries in sub-Saharan Africa. In terms of the sector, from the background readings, it is noted that this study is one of the first empirical studies to have been done with regard to community participation in the health sector in the country. Geographically, the study area is one of the newest and is also the poorest district, both within the Ashanti region and the country as whole. It is also one of the districts that still struggles with the prevention and control of the disease as a result of lack of resources. In essence, the outcomes of this study could help the malaria planning unit and all of the other stakeholders involved in the control programme activities, to enhance their intervention strategies relating to the extent of the community's involvement in programme activities.

## The definition of community participation

Although the notion of community participation has been known to be significant in health programme activities, there is still a dispute over its actual meaning and the way it should be assessed.^16,17^ It has, however, generally been accepted that community participation is not only the means by which communities become aware of the challenges facing them but also a better instrument for empowering and facilitating better living conditions for those who are underprivileged within the society.^18,19,20^ The above characterisation highlights the importance of the power associated with participation,^21^ which helps community members to be able to identify what they need, make decisions and develop the means of attaining such desires.^22,23^ In so doing, they can take control of their own health and wellbeing. This eventually helps them to build their own capacity to sustain local development rather than having to rely on external agents.^22,23,24^

In addition, it has also been argued that engaging communities provides two contrasting but useful definitions of community participation, namely, ‘participation as a means’ and ‘participation as an end’.^25^

*Participation as a means* implies that the way people take part in programme activities is characterised by a situation where the goal or objective (not known to all the members) has already been set for them by those in higher positions.^26^ Under these circumstances, participation becomes a tool through which the available resources of the community are used in order to attain the desired goal. This kind of participation often appears to be short lived and usually fails to make use of the results of the strenuous efforts put in by the various community members.^22,24^ In this way, participation is seen as something imposed by a higher authority as it does not reflect the original ideas of the local community. Rather, it becomes a device employed by powerful organisations, such as governments, looking for involvement as *a means* of utilising community resources such as local knowledge in order to ensure programme effectiveness.^23,27,28^

*Participation as an end*, however, has the tendency to be a long-term process, contributing toward building and fortifying the self-capabilities of the community members and enabling them to become more involved in health programmes. Participation in this sense helps to achieve goals such as social justice, equity and democracy.^29^ In this situation, the source, the form of participation and the process are understood and initiated by the community members’ themselves, which enhances integration and empowerment.^30^ The underlying idea is that not only are the community members’ independence and management skills enhanced, but their capacity to make decisions that have a direct impact on their lives is also nurtured.^31,32,33^

In effect, it can be argued that, through participation, communities can either be led by others to recognise their local health challenges and develop a method of solving them, or they can be empowered to address their own problems and to realise the basic healthcare needs for all of the members (*an end*).^34,35,36^.

## Community participation and Arnstein's ‘ladder’ framework

The main focus of Arnstein's argument on the issue of community participation is on power. According to Arnstein, citizen participation is a process which demonstrates not only the way in which power is shared but also how those that are marginalised in society are allowed to be involved in the decisions that affect them. It is, therefore, a mechanism to allow for the underprivileged to take part in the necessary reforms that can bring about change in their society and to allow them to have a share in the wealth of their community.^15^

**FIGURE 1:**
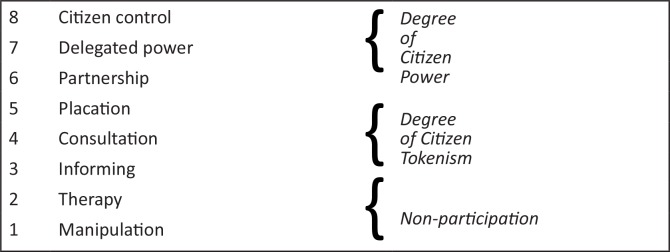
Arnstein's ladder of participation. *Source*: Arnstein 1969^15^

In measuring community participation, the various degrees to which a community has connections to power was compared to a ladder.^15,27,32^ Based on this analogy, Arnstein suggested a framework with three main levels, as depicted in Figure 1.

The lowest of all the levels represents a position where no participation takes place at all.^37^ After this level, the next levels are characterised by situations wherein those who are marginalised in the society are informed and consulted.^16^ Next to these levels are superior degrees which allow community members to be able to influence decisions in various ways through, for example, collaboration with those at a higher level of authority. The final level represents the highest position, where the members have power over the decision-making process.^25,32,36^

In essence, the framework demonstrates the way in which power is redistributed and its significance lies in the standard that is employed in order to make a distinction between those who are at a higher position and the ordinary people found at the lower level of society. At the lower levels, Arnstein^15^ argues that there are certain types of participation that provide an opportunity to the community members to either support or be informed about decisions which, by and large, have already been made. Under such circumstances, community participation becomes temporal and is sometimes only ceremonial. In addition, communities are allowed to participate, for example, in developed health programme plans and activities, so as to ensure legitimacy, gain support and prevent future criticism or sabotage. However, at the more advanced (ultimate) levels, the kind of participation that takes place can often be seen as potentially empowering. Participation at these levels allows the community the ability to mobilise and transform themselves and ensures that access to resources and services become relatively more easy.^27,38,39^

## Research methods and design

The method for the study was a qualitative exploratory approach involving in-depth interviews and focus group discussions (FGDs) with local health officials and community members at the district, subdistrict and village levels. The data were collected from October 2009 to February 2010 in the AAS district in the Ashanti region. The district occupies a total land area of 124 km^2^ and its capital is Mankranso. It has a total population of 174 612, with an adult population of approximately 100 000.^37^ The adult literacy rate is 41.1% and, to put it into the national context, the district literacy rate of 41.1% is below the national literacy average rate of 57.9%.^11^ In terms of occupation, about 80% are farmers as against the national average of almost 60% of the labour force who are engaged mostly in cocoa, vegetables, plantain and maize farming. However, it is worth mentioning that the choice of this district was for a practical purpose. In fact, whilst all of the districts in Ghana are malaria-endemic districts and therefore any of them could have been chosen, the selection of Ashanti and the AAS district was based on two main reasons: firstly, it was, because of practical considerations (such as availability of background information and familiarity with the local language), an available research network; and secondly, the opportunities for cooperation from the policy actors and the communities were assured.

In total, 80 individual in-depth interviews, including four FGDs (involving 46 participants with approximately 12 participants per group), were conducted. Two groups of interviewees were identified. The first group selected was from the local public health staff (20 interviewees), whilst the second was from within the communities. The latter group included community residents and a number of village health volunteers in malaria control programme activities. The sample size included both genders whose ages ranged from 18 to 70 years. Individual interviews lasted for approximately 45 minutes, whereas the FGDs took about two hours.

### Data analysis

In analysing the data, all the audio-taped interviews were first transcribed from the local language (Ashanti) to English and this was done with care so as to maintain the original meaning of the dialogue that took place.^40,41^ In achieving this, three main guiding principles were used, in line with other qualitative research enquiries. Firstly, the full interviews were read through with the aim of identifying the common themes. Secondly, each sentence was examined for the purpose of identifying the main idea behind the sentence and to give it a name or concept, that is to say, there were certain statements that could be understood only in a Ghanaian or local context and these thus needed to be interpreted for the researcher. In this way, by asking for the local explanations of certain concepts, the researcher's subjectivity was controlled during this creation process. Finally, concepts that were alike were put together to form categories which were more specific and could be generalised.^42,43^ Overall, two major categories were identified and these included the local health officials’ and the community members’ perspectives on the extent to which they participate in the control policy process.

## Ethical considerations

Permission to undertake this research was approved by the University of Southampton Ethics Board and, subsequently, by the Ministry of Health in Ghana. All participants were assured of confidentiality and the whole project was explained to them verbally, often in their own local language. Consent forms were also given to participants who agreed to take part.

## Results

### Characteristics of study population – The socio-demographic characteristics of the participants

Of the 80 participants who took part in the interviews, 44 (55%) were women and 36 (45%) were men. The overall mean age was 39.5 ± 1.5 years (ranging between 18 and 70 years) with the highest number of participants found in the 30–49 year age group (45%). Seventy percent (*n* = 56) of the participants were married and in terms of education, only 11% (*n* = 5) of the women and 15% (*n* = 5) of the men had education equal to senior secondary school or above. All of the rest of the women had education either up to junior secondary school (*n* = 30; 69%) or no education at all (*n* = 9; 20%), whilst 75% (*n* = 27) of the men had education up to junior secondary school and 10% (*n* = 4) had no education at all. With regard to occupation, more than two-thirds of the interviewees were farmers (74%), whilst just over one-quarter (*n* = 21; 26%), including government officials, were either salaried workers or local people who owned their own business (e.g. a shop). In essence, most of the interviewees were poor rural farmers.

### The opinions from local health officials on community participation

In using Arnstein's framework, the results of this study demonstrated that there was no real community participation in the NMCP (National Malaria Control Programme) policy-making process in the study site. Evidence in the study, so far, has shown that the mechanism used for public participation in the NMCP policy-making process could be described, in the words of Arnstein, as being ‘tokenistic’ with ‘informing’ and ‘consultation’ (p. 217)^15^ being the types of participation.

This description was the result of the opinions expressed by the most of the local health staff who were interviewed. During the interview, it was found that although some of the officers (*n* = 5; 25%) believed that there had been participation, the majority of the interviewed officers (*n* = 15; 75%) expressed their doubts regarding the extent of community participation during the planning process. According to the views shared by the majority of interviewees, despite the fact that the local health representatives were often informed about the planning process, they had no power to influence any decision that would be made during meetings, as was reflected in most of the arguments made by some of the participants during the interviews:

‘We consult them when it is necessary and it is not always the case that their decisions are acted upon in the process.’ (P3, Male, 38, District malaria control officer)

‘Although community members are those closer to the problem especially malaria, we can take decision without them, or we usually call on them but in the end, their views do not count very much.’ (P5, Female, 26, Community health nurse)

‘I am not surprised that the community members are side-lined when it comes to the final decision on priorities because it is the government who has the resources. It is a pity but that is the reality of the situation.’ (P11, Male, 35, Non-governmental organisation [NGO] worker)

Firstly, these arguments confirm that the final outcome of the malaria control programme activities do not represent the views of all those involved. This indicates that the use of ISC as a mechanism to enhance community participation in (e.g. NMCP) policy-making process is not effective. Secondly, the general picture that one can get from these arguments is that when it comes to the planning process, the inclusion of community members’ priorities, as well as the chances to explore the cultural significance of their views, is lacking. Such limited participation by the communities‘ can lead to the neglect of important sources of lay knowledge particularly top local decisions’ (p. 79).^44^ This raises a question as to whether community participation in NMCP at the local level is a means or an end. From the above analysis, it is fair to say that the community participation in the malaria control policy-making process may be described aptly as a tool for the attainment of the needed goal of the health authorities through the use of the community's own resources (*a means*). This is in contrast with having communities themselves becoming more involved in developing their own capabilities in order to achieve the desired goals (*an end*) without being dictated to by the health officials.^45,46,47^

However, the few officials who believed that participation *had* taken place argued that, faced with the constraints within the various districts, all of the institutional mechanisms that had been put in place at the community level were the ideal way to consult the community members. Arguments made in support of this claim reflected the views that the complex nature of the concept ‘participation’ had made the issue more difficult to handle. This can be seen from the following statements:

‘Participation is a “subtle concept” and it can be interpreted by different people in various ways, so it all depends upon how one views it.’ (P2, Female, 41, District health manager)

‘[*I*]f we do not communicate to [*sic*] them, how can we even get their views? They have representatives and all of them are invited to attend some of the general meetings.’ (P12, Male, 29, District health worker)

‘We expect their representatives to consult their local people before they come and we believe that whatever they present here reflect [*sic*] the views of the community.’ (P18, Female, 33, Local health education officer)

‘We have selected community health committee members who are frequently consulted. These committee members act as the voices for the community. So, yes, they are part of the process although we make the final decision.’ (P10, Female, 28, District clinical nurse)

The above arguments suggest that through decentralisation, provision has been made for the community members to participate in the planning process, of which authorities at the higher level are aware. To these officials, with this decentralisation policy, there is an opportunity given to the community members to participate in the health (e.g. NMCP) decision-making process through their local representatives.

### Local residents’ views on the extent of their participation

Similar to the majority of health officials, almost all of the community members (*n* = 46; 76%) also held the view that despite the fact that members have been participating in the process in various forums (e.g. general meetings, workshops, interviews and, indirectly, through their local health committees), the level of participation leaves much to be desired. For most of the members (*n* = 46; 76%), the participation of the community has been a reaction to what the health planners would like them to do. This was reflected in most of the arguments put forward by the majority of those who voiced their opinions during the FGDs. For example, one participant made the following statement:

‘[*T*]hey always come here to tell us what they intend to do but how they arrived at such decision is not something we have any means to know.’ (P57, Male, 55, Local chief)

Others added:

‘[*I*]t is all good for them to contact us after they have taken a decision and come to seek our support but what I would have liked is to have us during the time of decision taking [*sic*].’ (P77, Male, 23, Local youth organiser)

‘Although we have our community health committees, they only bring us into the discussion when they have made a decision on how they want us to give our support. I find it wrong.’ (P81, Female, 35, Head of women's traders association)

‘[*W*]e are only to obey what they tell us to do but nobody comes to say, this date or that date we want you to come so we can all decide on what is best for you.’ (P55, Female, 33, Zonal health committee member)

‘Often when the health officials who come here … I think they purposely come here to make us know what they intend to do which is not the same as asking for our opinions.’ (P60, Male, 44, Elected zonal assemblyman)

However, from the perspective of a minority group within the interviewees (*n* = 14; 24%), it was noted that although they did admit that there was only consultation, they were satisfied with the type of participatory approach that was adopted. During the interview, various assertions were made by the interviewees. For example, one of the farmers said:

‘Definitely, we are not considered at the initial stages of the planning process, but when it comes to health, it is good that doctors, nurses do the planning.’ (P62, Male, 61, Local chief cocoa farmer)

In addition, a local taxi driver also expressed his concern, saying:

‘I think we do need these experts to decide for us, especially health complex issues that affect our lives like malaria. I think it will be wrong to let us decide on our own.’ (P75, Male, 31, Local taxi driver)

Finally, at the time of the FGDs, another participant said:

‘They are trained to do that job and we are only to support them. So consulting us alone for me is okay.’ (P59, Male, 40, Local shop owner)

The views expressed above by the community members reflect two divergent but important fundamental views regarding how the concept of community participation is defined in the literature. Firstly, there were those (*n* = 14; 24%, i.e. minority views) who acknowledged that community participation had been minimal, but nonetheless accepted it to be a step in the right direction. For them, if there was any participation at all, it should be as a reaction to the wishes of the health experts. This perception of participation agrees with the narrowest level of Rifkin's^24^ idea of health which is considered to be a condition where there is no existence of illness (the medical approach). In this context, community participation is described as being a process whereby community members perform certain tasks, such as providing a healthy environment within a community, under the directives of health experts.^24^ What this means is that community members are only service users and must therefore follow the orders provided by the professionals without taking part actively in policy issues that affect them.^46,47^

Thus, with regard to malaria, some members within the community wanted to be passive when it comes to decisions that affect their own health. They preferred, instead, to leave such a task in the hands of the health experts whilst following their orders.

In contrast, the second group, (*n* = 46; 76%, i.e. majority views) saw community participation in a wider context and perceived community participation to be the community members’ involvement in NMCP policy decisions without necessarily resorting to the dictates of the health experts. This way of seeing community participation is consistent with the second approach of Rifkin,^24^ which is called the *health service approach*. With this approach, community participation is viewed in the wider context as ‘the mobilisation of community people to take an active part in the delivery of health services’ (p. 241).^24^

To this group of people, decision-making regarding malaria should not be left solely in the hands of health professionals; instead, the community members should also be involved. This is in contrast to the minority group who argued that because of the complex nature of health and, for that matter, malaria, decisions should not be left to anyone other than the health professionals. This seems to suggest that the second group's majority opinion exemplifies the belief that the definition of health should not be merely the absence of disease (the medical approach), but should rather have the broader meaning of the word, which involves ‘the physical, mental and social wellbeing of the individual’.^48^

## Discussion

This study finding reflects the extent to which community members are given the chance to voice their concerns regarding NMCP activities in the AAS district of the Ashanti region in Ghana. There have been underlying variations in the definitions of community participation by those involved in malaria control programme activities which have given rise to both convergent and divergent perceptions.

On the one hand, the perspective of the local officials is there *has* been community participation. To them, as long as community representatives from the district health sector are invited to attend meetings, the community members are participating in the NMCP process. It is felt that these representatives represent the interests of the community and therefore have the opportunity to bring their communities’ concerns to the discussion table.

On the other hand, there are those who think that there has been *limited* involvement of communities in the planning process. Some interviewed local officers agreed with the idea that when community groups are invited to participate, the ultimate real decisions are still in the hands of the state officials. There is therefore none of the empowerment and effective participation which the health policy of decentralisation purports to achieve.^49,50^

This raises a question as to whether community participation in NMCP at the local level is employed as a *mean*s or an *end*. From the above analysis, it is fair to say that the concept of community participation offers no opportunity to community members to attain real power at the local level policy-making processes. Instead, the community members, including their representatives, only play, in the words of Arnstein, a ritual role.^15^ They are not permitted to have control over their own health situation but rather are coopted into direct forms of participation which are in essence inactive, passive and, eventually, a convenient way of controlling participation^44^ – the participation is not really intended to have any significant input from the community members toward the decision-making process.

From the context of Arnstein's ladder of participation framework,^15^ such participation can be regarded as tokenistic and the highest quality of participation is at consultation levels. This only guarantees the provision of information and consultation on issues and it neither leads to community empowerment nor ensures direct incorporation of input from the communities into the policy-making process. This implies that the communities are only used as tools (means) for achieving policy goals rather than being the actual decision makers (an end) regarding their local health problems.

Overall, the findings have demonstrated some key issues which demonstrate that successful implementation of the ISC strategy in the malaria control planning process has not yet taken place. In essence, despite the fact that the process and the right to participate may be devolved to the local level, the community's influence, which could mould and challenge policy development, as well as supervise the outcomes, is still in the hands of the health authorities. This is in contrast to the national health policy statement which is aimed at ensuring the empowerment of the community. However, what the health officials fail to realise is that participation is not merely about having the opportunity to consult or being informed about policy outcomes or attending meetings, but also about having the power to control inputs which would otherwise have been controlled by others who may or may not address the communities’ priorities.^23^

This finding also reflects the power relationship between the community (weak) and the health experts (strong). To adopt this kind of ‘induced’ form of participation means that communities only accept ideas on local development that have been developed for them by health authorities instead of the members developing these ideas by themselves.

Community members thus only participated in the NMCP policy-making process when the health authorities had to inform them about certain activities rather than engaging with them regarding policy plans. Consequently, the community members had no power to influence negotiations and, under this type of participation, Arnstein has argued that community participants seem to be participating in policy process solely to give backing to government's decisions.^15^ In addition, the health authorities only consulted the community members by organising general community meetings, but there was no guarantee that their suggestions and input on priorities would be considered or acted upon in the final priority selection. This kind of participation, in the words of Arnstein, is regarded as ‘placation’.^15^ In effect, participation by the communities could be deemed merely ceremonial, seeing as the policy-making process still bears all the features of a top-down system of decisionmaking.^49,50,51^

This is in contrast to Arnstein's idea that citizen participation involves the exchange of power through a mechanism which could promote the interests of communities in the policy processes. Participation that does not give power to the local community members can be considered to be a fruitless exercise which leads the members nowhere and ‘only maintains [*the*] status quo’ (p. 219).^15^

On the whole, using Arnstein's ladder of participation, it is fair to say that rather than being a reality, the level of community participation in the NMCP policy-making process could be termed as a myth characterised merely by provision of information and consultation.

### Limitations

Although the methods employed in this study were useful, the author acknowledges that this study is not without its limitations. One potential limitation is that the responses to questions were based on individuals’ own perceptions and could therefore be said to be subjective, rather than objective. However, this was addressed through the use of the triangulation method which involves cross-checking with others for further details as well as finding out more from the existing records and literature. Using such a method to establish the validity of the information received helped with a better comprehension of the issue under study.^52^

The second limitation was that the study was confined to only one district in Ghana and, as such, the results could not be generalised since other people in other districts could have their own opinions on the subject.

## Conclusion

This article has considered the extent to which communities get involved in malaria control policy issues in the AAS district in Ghana. The study used Arnstein's ladder of participation framework to measure the level of participation. The findings showed that although the community members were allowed to take part at the district level policy-making process of malaria control, there was no power offered to the local residents. Indeed, their participation has been limited purely to information, consultation and placation throughout the process. As such, whilst the idea of participation is rooted in both international and national health policy documents, there is little explicit practical operation thereof in terms of power sharing.

This study's results, therefore, raise concerns over the effectiveness of the ISC policy strategy of national control programme to enhance community participation. Whilst it can be argued that the government is committed to controlling malaria, the same cannot be said about its commitment in promoting community participation through the ISC strategy. Having no clear and effective mechanisms for the empowerment of community members seems to put the achievement of the ideals of Alma Ata of 1978^48^ and the Millennium Development Goals^53^ beyond the reach of the Ghanaian government at present.
